# Assessment of Inhibition of Biofilm Formation on Non-Thermal Plasma-Treated TiO_2_ Nanotubes

**DOI:** 10.3390/ijms24043335

**Published:** 2023-02-07

**Authors:** Min-Kyung Ji, Seon-Ki Lee, Hee-Seon Kim, Gye-Jeong Oh, Hoonsung Cho, Hyun-Pil Lim

**Affiliations:** 1 Dental 4D Research Center, Chonnam National University, 33 Yongbong-ro, Buk-gu, Gwangju 61186, Republic of Korea; 2 Department of Prosthodontics, Daejeon Dental Hospital, Wonkwang University, 77 Dunsan-ro, Seo-gu, Daejeon 35233, Republic of Korea; 3 Department of Prosthodontics, School of Dentistry, Chonnam National University, 33 Yongbong-ro, Buk-gu, Gwangju 61186, Republic of Korea; 4 Biomedical Evaluation & Research Center, Chonnam National University, 33 Yongbong-ro, Buk-gu, Gwangju 61186, Republic of Korea; 5 School of Materials Science & Engineering, Chonnam National University, 77 Yongbong-ro, Buk-gu, Gwangju 61186, Republic of Korea

**Keywords:** TiO_2_ nanotube, heat treatment, non-thermal plasma treatment, peri-implantitis, biofilms

## Abstract

Peri-implantitis is an inflammatory disease similar to periodontitis, caused by biofilms formed on the surface of dental implants. This inflammation can spread to bone tissues and result in bone loss. Therefore, it is essential to inhibit the formation of biofilms on the surface of dental implants. Thus, this study examined the inhibition of biofilm formation by treating TiO_2_ nanotubes with heat and plasma. Commercially pure titanium specimens were anodized to form TiO_2_ nanotubes. Heat treatment was performed at 400 and 600 °C, and atmospheric pressure plasma was applied using a plasma generator (PGS-200, Expantech, Suwon, Republic of Korea). Contact angles, surface roughness, surface structure, crystal structure, and chemical compositions were measured to analyze the surface properties of the specimens. The inhibition of biofilm formation was assessed using two methods. The results of this study showed that the heat treatment of TiO_2_ nanotubes at 400 °C inhibited the adhesion of *Streptococcus mutans* (*S. mutans*), associated with initial biofilm formation, and that heat treatment of TiO_2_ nanotubes at 600 °C inhibited the adhesion of *Porphyromonas gingivalis* (*P. gingivalis*), which causes peri-implantitis. Applying plasma to the TiO_2_ nanotubes heat-treated at 600 °C inhibited the adhesion of *S. mutans* and *P. gingivalis.*

## 1. Introduction

Bacterial colonies that cause periodontitis are formed on the surface of dental implants within two weeks after being placed and exposed to an oral environment. Subgingival bacterial flora is established within four weeks, resulting in tissue inflammation and, thus, dental implant failure [1]. Since dental implants, unlike natural teeth, do not have any blood vessels or nervous tissues, patients do not feel any particular pain associated with peri-implantitis, and the inflammation is highly likely to spread to bone tissues and result in bone loss [2]. Therefore, it is essential to increase the bioactivity of the surface of the implants and suppress the formation of biofilms in order to maintain dental implants.

Various attempts have been made to modify the surface of dental implants to suppress bacterial growth and improve the adhesion of osteocytes [3]. Anodization is one method of modifying the titanium surface [4]. A thin, rough, porous amorphous TiO_2_ oxide film is electrochemically created on the titanium surface to modify its physical properties. Once amorphous TiO_2_ nanotubes are formed on the surface of the titanium implants via anodization, their surface area is expanded, and cell adhesion, proliferation, and differentiation are improved, which was reported to increase bone–implant integration, accelerate bone formation and reduce treatment times [5,6,7]. Peng et al. [8] reported that the initial adhesion and colonization of *Staphylococcus epidermidis* on TiO_2_ nanotubes were significantly reduced when compared with polished or acid-etched titanium. In addition, heat treatment of the titanium surface changes the crystal structure of the TiO_2_ films and suppresses the adhesion of bacteria without inhibiting the activity of cells [9,10]. Del Curto et al. [11] reported that the adhesion of bacteria was inhibited on the titanium surface by modifying its amorphous oxide film into an anatase crystal structure through heat treatment without inhibiting the activity of cells. However, in another study, osseointegration was further improved on a rutile crystal structure surface [12]. Furthermore, opinions are divided on whether anatase or rutile is a better TiO_2_ oxide film for titanium implants. The adhesion of bacteria and the activity of cells are affected not only by the physical properties of dental implants, such as surface structure and roughness, but also by chemical properties, such as surface-free energy and hydrophilicity [5,6,13]. 

The chemical properties of dental implants, such as surface-free energy and hydrophilicity, play a critical role in the interaction between osteocytes and proteins and thus improve the treatment of cuts in the early stage of treatment [14,15]. Sawase et al. [16] found that the surface of dental implants with improved hydrophilicity showed better osseointegration than that of general implants. These results indicated that the chemical properties of the surface of dental implants are more important factors for the activity of cells than their surface structure. 

Plasma is a gas with an energy that is loosely and partially ionized [17] and is used to sterilize the surfaces of dental equipment and biomaterials and to modify the surfaces of implants [18,19]. By applying plasma, the oxide layer on the surface of implants is modified due to the chemical properties of the applied gas without any change in the surface structure, which results in chemical changes, such as hydrophilicity [20]. Yoo et al. [21] reported that the shape of bacterial colonies cultured on the surface of titanium treated with plasma was changed and that over 80% of bacteria were killed. In addition, Duske et al. [13] found that plasma decreased contact angles and thus increased hydrophilicity, improving the early adhesion of osteoblasts and osseointegration. 

Even though the chemical properties of the surface of dental implants are as important as their surface structure in inhibiting the adhesion of bacteria and the activity of cells, studies have been conducted only to evaluate changes in the surface structure and roughness of dental implants. Authors have been mostly focused on changes in the activity of cells and osseointegration after applying atmospheric pressure plasma to the surface of implants. However, there has been no study published on the inhibition of peri-implantitis. Moreover, previous studies on biofilm inhibition of TiO_2_ nanostructures use antimicrobial agents (e.g., Ag, Au) on TiO_2_ nanostructures [22,23]. This study created TiO_2_ nanotubes on the titanium surface to change the physical properties of the surface of dental implants. In addition, heat treatment and atmospheric pressure plasma were applied to change the chemical properties of the surface of dental implants.

This study examined the effects of heat and plasma treatment on the surface of TiO_2_ nanotubes on biofilm formation.

## 2. Results

### 2.1. Surface Characteristics

#### 2.1.1. Contact Angles

**Figure 1:**
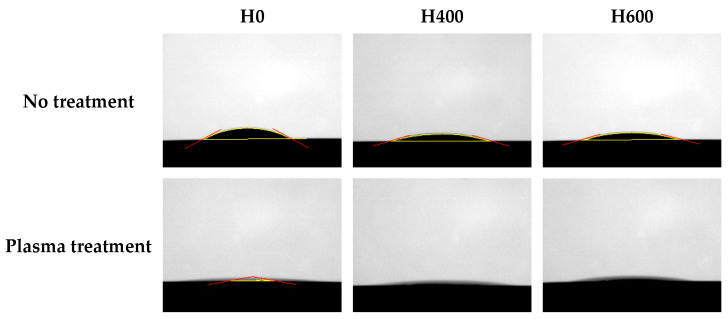
The water contact angle of the samples. The yellow line indicates the solid-liquid interface and the liquid-fluid interface. The red line indicates the contact angle.

The contact angle of the non-heat-treated (H0), 400 °C heat-treated (H400), and 600 °C heat-treated (H600) samples was 19.5 ± 2°, 14.2 ± 2°, and 18.0 ± 2°, respectively. After the plasma treatment, the contact angle of all groups was reduced. The contact angle of the non-heat-treated plasma-treated samples (H0P) was 3.3 ± 6°, and the contact angle of the 400 °C heat-treated plasma-treated samples (H400P) and the 600 °C heat-treated plasma-treated samples (H600P) was reduced to the point that could not be measured (Figure 1). 

#### 2.1.2. Surface Structures

**Figure 2:**
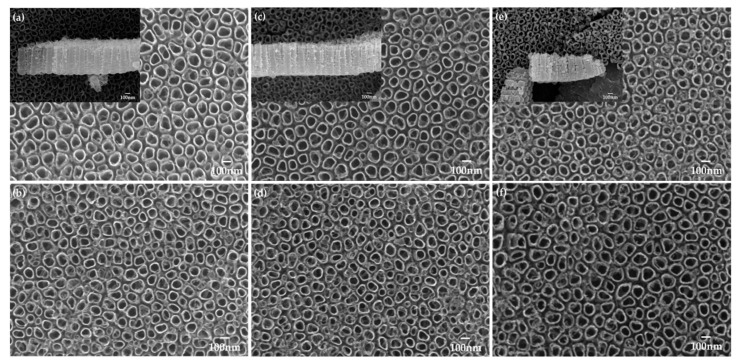
Field emission-scanning electron microscopy images on (**a**) H0, (**b**) H0P, (**c**) H400, (**d**) H400P, (**e**) H600, and (**f**) H600P samples (magnification = 50,000). The mini-subfigures of the left-top corner indicate the height of TiO_2_ nanotubes.

There were no changes in the height and surface structures of the TiO_2_ nanotubes after the heat treatment at 400 °C. However, when the samples were heat-treated at 600 °C, the diameter was decreased to 91.2 ± 1 nm, and their height was reduced to 498.0 ± 7 nm (Figure 2, Table 1). There was no change in the surface structure of the samples before or after the plasma treatment.

#### 2.1.3. X-ray Diffraction (XRD) Analysis

**Figure 3:**
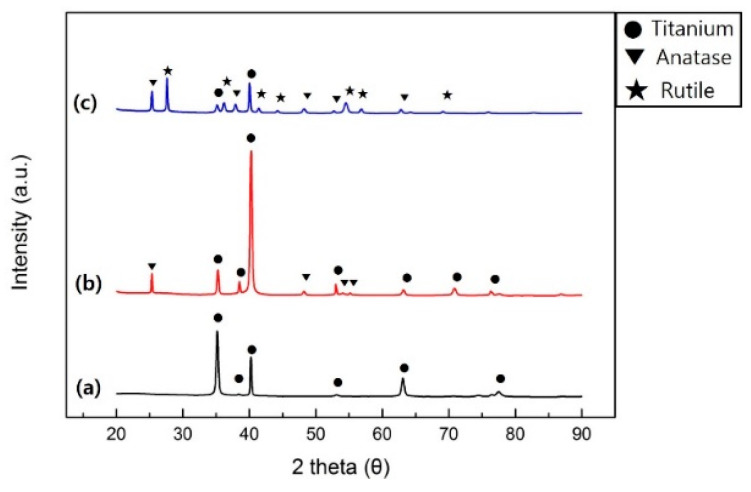
X-ray diffraction patterns of specimens. (**a**) H0, (**b**) H400, and (**c**) H600 samples.

Figure 3 shows the X-ray diffraction patterns of samples. The H400 samples showed titanium reflections at (010), (002), (011), (012), (110), (013) and (112), and TiO_2_ anatase reflections at (011), (020), (015), and (121). The H600 samples showed a different peak pattern; however, the same reflections were observed as the H400 samples plus new TiO_2_ rutile reflections at (110), (011), (111), (120), and (121). The analysis results of H600 samples showed that the titanium, TiO_2_ anatase and rutile crystal structures were mixed.

#### 2.1.4. X-ray Photoelectron Spectroscopy (XPS) Analysis

**Figure 4:**
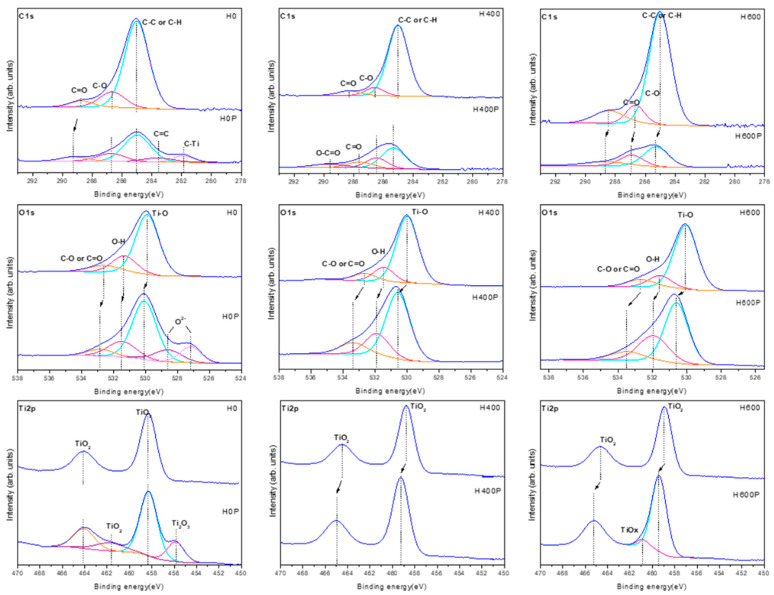
XPS high-resolution spectra of C1s, O1s and Ti2p on samples.

Figure 4 shows the XPS high-resolution spectra of C1s, O1s and Ti2p on samples. Out of the three C1s peaks (285.0 eV, 286.7 eV, and 288.7 eV) detected in the H0 samples, the 285.0 eV peak represents C-C or C-H bonded carbon, the 286.7 eV peak represents C-O bonded carbon, and the 288.7 eV peak represents C=O bonded carbon. Out of the three O1s peaks (529.9 eV, 531.3 eV, and 532.6 eV), the 529.9 eV and 531.3 eV peaks are associated with Ti-O and O-H bonds, while the 532.6 eV peak is associated with C-O or C=O bonds. In the case of Ti2p, two distinct peaks were observed at 464.1 eV and 458.3 eV, representing TiO_2_. 

After heat treatment, the O1s peaks (530.0 eV, 531.5 eV, and 532.6 eV) and the Ti2p peaks (464.5 eV and 458.7 eV) of the H400 samples, as well as the O1s peaks (530.1 eV, 531.6 eV, and 532.7 eV) and the Ti2p peaks (464.7 eV and 458.9 eV) of the H600 samples were slightly shifted to a higher bonding energy.

After plasma treatment, the C1s peaks were reduced, and the low peaks between 292.1 eV and 281.2 eV were slightly shifted to a higher bonding energy. Another C1s peak was observed in 283.6 eV and 281.9 eV, which are associated with C=C and C-Ti. Two additional O1s peaks (528.6 eV and 527.2 eV) were observed, which represent O^2−^. The Ti2p1/2 and Ti2p3/2 peaks were observed at 461.7 eV and 456.1 eV, respectively, which are associated with TiO_2_ and Ti_2_O_3_. Another C1s peak in the H400P samples was observed at 289.7 eV, which is associated with O-C=O. Moreover, another Ti2p peak in the H600P samples was observed at 460.9 eV, which is associated with TiOx, a titanium compound.

### 2.2. Assessment of Capacity to Inhibit Biofilm Formation

#### 2.2.1. *Streptococcus mutans*

**Figure 5:**
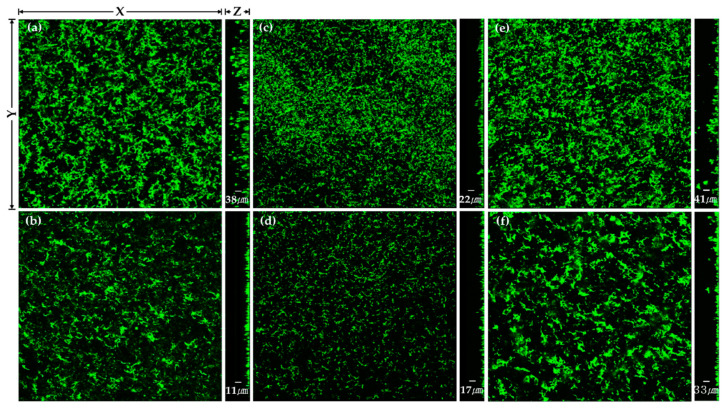
CLSM image of *Streptococcus mutans* biofilm on (**a**) H0, (**b**) H0P, (**c**) H400, (**d**) H400P, (**e**) H600, and (**f**) H600P samples. Green fluorescence indicates viable cells.

The changes in the thickness of *S. mutans* biofilms after the heat and plasma treatments to the TiO_2_ nanotubes are shown in Table 2 and Figure 5. 

Compared with the H400 samples, the H0 and H600 samples showed a significantly smaller thickness of *S. mutans* biofilms (*p* < 0.017). There were statistically significant decreases in the thickness of *S. mutans* biofilms in all the groups after plasma treatment (*p* < 0.05). 

#### 2.2.2. *Porphyromonas gingivalis*

**Figure 6:**
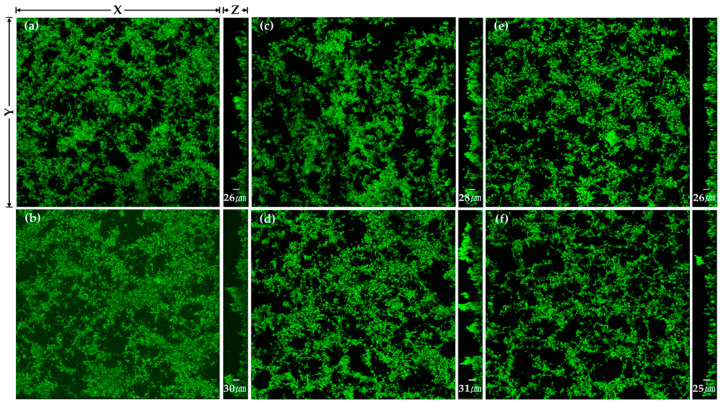
CLSM image of *Porphyromonas gingivalis* biofilm on (**a**) H0, (**b**) H0P, (**c**) H400, (**d**) H400P, (**e**) H600, and (**f**) H600P samples. Green fluorescence indicates viable cells.

The changes in the thickness of *P. gingivalis* biofilms after the heat and plasma treatments to the TiO_2_ nanotubes are shown in Table 3 and Figure 6.

There were no statistically significant differences in the thickness of *P. gingivalis* biofilms after the heat treatment (*p* > 0.017). There were also no statistically significant differences in the thickness of *P. gingivalis* biofilms after the plasma treatment (*p* > 0.05).

## 3. Discussion

**Figure 7:**
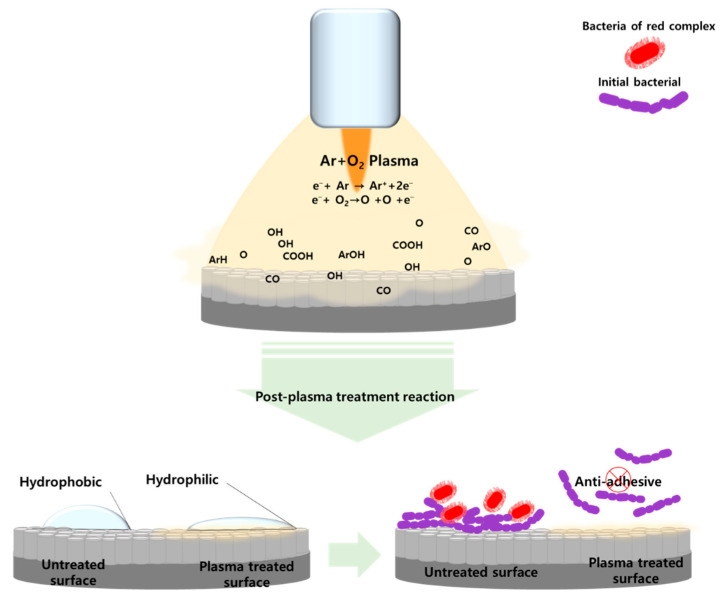
Schematic of a summary of possible events on the plasma-treated TiO_2_ nanotube surface.

It is essential to inhibit the adhesion of bacteria to the surface of dental implants to improve the adhesion between the surface and surrounding tissues. In this study, TiO_2_ nanotubes were formed on the titanium surface to increase its surface area. The TiO_2_ nanotubes were heat-treated under different conditions to modify the structure of the TiO_2_ oxide films. In addition, atmospheric pressure plasma was applied to the TiO_2_ nanotubes to change their surface energy and hydrophilicity and, thus, inhibit the formation of biofilms. Figure 7 shows a schematic of a summary of possible events on the plasma-treated TiO_2_ nanotube surface. Recently it has been reported that the adhesion and proliferation of osteoblasts were increased on the surface of materials that had nanotube structures, and studies have been conducted to create nano-level structures on the surface of materials [24,25,26]. Electrochemical anodization can be used to create nano-level tubes on the surface of titanium, and the structure of nanotubes can be changed by controlling certain variables, such as the composition of electrolytes, temperature, and current density [27,28,29]. In this study, TiO_2_ nanotubes were created using the anodic oxidation method suggested by Kim et al. [30] to form a nano-level amorphous TiO_2_ oxide film on the surface of the titanium. 

Heat treatment of titanium can modify the crystal structure of the TiO_2_ oxide film. Park et al. [31] reported that heat treatment of 400 °C changed the phase of the amorphous TiO_2_ into anatase TiO_2_ and that temperatures over 600 °C changed the phase to an intermixed population of anatase and rutile phases with the TiO_2_ rutile phase forming a major X-ray peak. Therefore, in this study, the temperature of the heat treatment was set at 400 °C and 600 °C to assess the adhesion of bacteria to amorphous TiO_2_, anatase TiO_2_, and anatase/rutile mixed-phase TiO_2_. 

When TiO_2_, which has photocatalytic properties, is combined with light, it reacts with H_2_O and O_2_ and generates photo-generated holes (h+) that have a high oxidizing power, such as hydroxyl radicals (OH) and superoxide free radicals (O^2−^) [32]. These photo-generated holes (h+) interact with proteins, nucleic acids, and bacterial cell membranes and can damage the structures of bacteria [33,34]. However, the adhesion of bacteria after heat treatment showed different results with the two species of bacteria. The lowest adhesion of *S. mutans* was seen in the anatase TiO_2_ (H400), and the lowest adhesion of *P. gingivalis* was seen in the anatase/rutile mixed TiO_2_ (H600). The relative photocatalytic activity differed depending on the crystal structure. Zhang et al. [35] reported that anatase TiO_2_ usually exhibited higher photocatalytic activity than rutile TiO_2_. In addition to the crystal structure, the metabolism of bacteria is associated with complex factors that control the biointerface and bacterial behaviors. In this study, the adhesion of *S. mutans* onto anatase TiO_2_ seemed to be inhibited as the photocatalytic activities that affected the metabolism of *S. mutans* were more strongly dependent on the crystal structure. 

Fluoride ions seemed to be detected on every surface due to the anodic oxidation of electrolytes since fluorine in the anodized electrolytes remained on the surface of the TiO_2_ nanotubes. The heat treatment reduced the amount of residual fluorine, and the two species of bacteria observed in this study showed different results depending on the fluorine content *P. gingivalis* showed the lowest adhesion in the H0 samples where the fluorine content was the highest and statistically more adhesion in the H400 samples, where the fluorine content was the lowest. In comparison, *S. mutans* showed statistically less adhesion in the H400 samples, where the fluorine content was the lowest. Yoshinari et al. [36] reported that fluorine has two mechanisms that affect bacteria. One is that fluoride ions act as an enzyme inhibitor, affecting the metabolism of bacteria, and the other is that metal–fluoride complexes suppress proton-translocating F-ATPases. In *P. gingivalis*, the enzyme inhibitor mechanism of fluoride ions seemed to work more strongly. 

Chemical changes were observed on the surface of samples treated with plasma due to the composition of process gases [37]. The results of this study did not show any change in the surface structure of TiO_2_ nanotubes after the plasma treatment. Although there was no change in the surface structure, specimens treated with plasma showed a lower contact angle than those that were not treated. XPS analysis was performed to identify the functional groups that determine surface hydrophilicity and electric charge. The results showed that C-H and C-C peaks after plasma treatment were reduced and that O-H, O1s, and TiO_2_ peaks were increased. Previous studies have found that plasma treatment formed various oxygen functional groups on the surface of materials through chemical oxidation, which breaks C-C and C-H bonds on the surface and removes carbohydrates connected to C-H bonds [38,39]. It was also reported that oxygen, mixed for plasma treatments, attaches a hydroxy group (O-H) on the surface of titanium, and generates reactive oxygen species (ROS), which improves hydrophilicity [40]. 

In this study, a fluorescent nucleic acid staining method was used to assess the adhesion of bacteria after the plasma treatment, and the two species of bacteria showed different results. In *S. mutans*, the biofilm thickness on all surfaces was significantly reduced after plasma treatment regardless of heat treatment, while *P. gingivalis* did not show any statistically significant differences. It has been reported that bacteria adhere better to hydrophobic or nonpolar surfaces than to hydrophilic surfaces [41]. Hydrophobic surfaces increase the adhesion of hydrophobic bacteria [42], and hydrophilic surfaces increase the adhesion of hydrophilic bacteria [43]. *S. mutans,* used to test the changes in biofilm formation in this study, is a hydrophobic bacterial species [44], while *P. gingivalis* is hydrophobic and hydrophilic [45]. Therefore, the increase in the hydrophilicity of the TiO_2_ nanotubes after applying atmospheric pressure plasma can be attributed to the decrease in the adhesion of *S. mutans*, a hydrophobic species. 

This study assessed the adhesion of bacteria based on the thickness of their biofilms. If bacteria that adhere to the surfaces can be quantified, the adhesion of bacteria can be more accurately assessed. Only the reactions of two species of bacteria were observed in this study. Since various bacteria cause peri-implantitis, it is necessary to conduct further research on peri-implantitis using various species of bacteria or mixed bacterial colonies. In addition, this study was carried out using a single gas plasma and one treatment time; thus, it is also necessary to apply different gas processes and treatment times in future studies. The results of this study showed that heat and plasma treatments inhibited the adhesion of bacteria. However, it will be necessary to perform additional in vitro and in vivo experiments to verify the biostability of TiO_2_ nanotubes on the surfaces of dental implants.

## 4. Materials and Methods

### 4.1. Samples

Disk-shaped (diameter: 15 mm, thickness: 3 mm) commercially pure titanium specimens (ASTM Grade IV, Kobe Steel, Kobe, Japan) were wet ground (Labopol-5, Struers, Ballerup, Denmark) using #600 silicon carbide (SiC) paper. All specimens treated with heat and plasma were sterilized using ethylene oxide (E.O) gas.

### 4.2. Surface Treatment

#### 4.2.1. Anodic Oxidation

Anodic oxidation was performed using a DC power supplier (Fine Power F-3005, SG EMD, Anyang, Republic of Korea). An electrolyte mixture was prepared by adding 1 M phosphoric acid and 1.5 wt% hydrofluoric (HF) acid to distilled water. The titanium specimen was connected to the anode, and a platinum plate (3 mm × 4 mm × 0.1 mm) was connected to the cathode. The specimen and platinum plate were placed approximately 10 mm apart and dipped in the electrolyte mixture for one minute. After that, 20 V were applied to the specimen and platinum plate for anodic oxidation for 10 min.

#### 4.2.2. Heat Treatment

The heat treatment was performed using a sintering furnace (DUOTRON PRO ex-6100, ADDIN Co., LTD, Suwon, Republic of Korea). Under an air atmosphere, the temperature was increased to 400 °C and 600 °C at a rate of 1 °C/min. The furnace was cooled after maintaining the temperature for one hour.

#### 4.2.3. Non-Thermal Plasma Treatment

The non-thermal plasma treatment was performed using an atmospheric pressure plasma generator (PGS-200, Expantech Co., Suwon, Republic of Korea) (Table 4). Ar and O_2_ gases were mixed in a ratio of 99% to 1%, and applied to the specimen at 300 W at a rate of 10 L/min. The distance between the plasma flame and the specimen was maintained at 5 mm. The specimen was rotated at 180 rpm, and the plasma flame was moved to the right and left ten times (12 s for one time) to evenly apply the atmospheric pressure plasma to the specimen. Thus, the plasma flame was applied for a total of 120 s (Figure 2).

### 4.3. Assessment of Surface Properties

#### 4.3.1. Contact Angles

Four microliters of distilled water were dropped onto the surfaces of the specimens to compare the changes in the hydrophilicity. The angle between the surface and the solution was measured after ten seconds using a video contact angle measuring device (Phoenix 300, SEO Co., Suwon, Republic of Korea). For each group, the angle of three specimens was measured and averaged.

#### 4.3.2. Surface Structures

The changes in the surface structure of the specimens were observed using a scanning electron microscope (FE-SEM S-4700, Hitachi Horiba, Tokyo, Japan).

#### 4.3.3. XRD Analysis

The changes in the crystal structure on the surface of the specimens were analyzed using an X-ray diffractometer (XRD) (X’Pert PRO Multi-Purpose X-ray Diffractometer, PANalytical, Almelo, The Netherlands). Diffraction was analyzed using X-rays of CuKα at a speed of 1.5°/min at an angle of 2θ ranging from 20° to 90°.

#### 4.3.4. XPS Analysis

The chemical compositions and bonds of specimens were analyzed using X-ray photoelectron spectroscopy (XPS, VG Mulrilab 2000, Thermo Scientific, Oxford, UK). The peak area values of each element detected were normalized and expressed as a quantitative ratio.

### 4.4. Assessment of Inhibition of Biofilm Formation

#### 4.4.1. Bacterial Culture

In this study, *Streptococcus mutans* (KCOM 1504), a Gram-positive aerobic bacterium associated with initial biofilm formation, and *Porphyromonas gingivalis* (KCOM 2804), a Gram-negative anaerobic bacterium that causes peri-implantitis, were used. The two species of bacteria were obtained from the Korean Collection for Oral Microbiology (KCOM, Gwangju, Republic of Korea)*. S. mutans* was cultured in a Brain Heart Infusion broth (BHI, Becton, Dickinson and Company, Sparks, MD, USA), and *P. gingivalis* was cultured in a Tryptic Soy Broth (TSB, Becton, Dickinson and Company, Sparks, MD, USA). Single colonies cultured on a solid medium were cultured in a liquid medium and grown to a concentration of 1.5 × 10^7^ CFU/mL. 

#### 4.4.2. Artificial Saliva Coating

Specimens were coated with artificial saliva to create an oral environment. The artificial saliva used in this study was prepared by adding 1% mucin (Mucin from porcine stomach, M1778; Sigma-Aldrich, St. Louis, MO, USA) to an adhesion buffer [46]. Three specimens per group were put in a 24-well plate, and the prepared artificial saliva was applied. The specimens were slowly stirred in the culture medium at 37 °C for two hours to coat the specimens with the artificial saliva. *S. mutans* was cultured in BHI at 37 °C (LIB-150M, DAIHAN Labtech Co., Namyangju, Republic of Korea), and *P. gingivalis* was cultured in anaerobic TSB at 37 °C (Forma Anaerobic System 1029; Thermo Fisher Scientific, Waltham, MA, USA).

#### 4.4.3. Bacterial Inoculation

After two hours, the artificial saliva was removed, and the specimens were dried for 15 min. Bacteria were inoculated on the surface of the specimens at a concentration of 1.5 × 10^7^ CFU/mL. *S. mutans* was cultured for 24 h, and *P. gingivalis* was cultured for 48 h.

#### 4.4.4. Fluorescent Nucleic Acid Staining Assessment

The biofilm formation was assessed using a green-fluorescent nucleic acid stain (SYTO 9^®^, Molecular Probes Europe BV, Leiden, The Netherlands). After culturing bacteria, the culture medium was removed. The specimens were carefully cleaned two times using phosphate-buffered saline (PBS) to remove bacteria that did not adhere. Afterwards, 200 μL of fluorescent dye (SYTO 9^®^:dH_2_O = 1.5 μL:1 mL) was injected onto the specimens. The plate was sealed with aluminum foil to block out light and was placed at room temperature for 15 min. After the reaction, the residual staining solution was carefully cleaned using PBS, and the surfaces of the specimens were observed using a laser confocal scanning microscope (Leica TCS SP5 AOBS/tandem, Leica, Bensheim, Germany). The thickness of the biofilms formed on the specimens was measured using an analysis program (Leica LAS AF software, Leica Microsystems, Bensheim, Germany).

### 4.5. Statistical Analysis

#### 4.5.1. Results of Heat Treatment

When the assumption of normality was met, the significance of the heat treatment results was tested using a parametric one-way analysis of variance (ANOVA) and Tukey’s post-hoc test. The significance level was set at a *p*-value less than 0.05. When the assumption of normality was not met, the results were statistically analyzed using a non-parametric Kruskal–Wallis test. The two groups were paired and tested using a Mann–Whitney U-test. Type I errors were corrected using Bonferroni’s method. The significance level was set at a *p*-value less than 0.017.

#### 4.5.2. Results of Plasma Treatment

When the assumption of normality was met, the significance of the plasma treatment results was statistically analyzed using a parametric independent *t*-test. When the assumption of normality was not met, the two groups were compared using a non-parametric Mann–Whitney U-test. The significance level was set at a *p*-value less than 0.05.

## 5. Conclusions

In this study, TiO_2_ nanotubes were formed on the titanium surface of dental implants to increase the surface area. TiO_2_ nanotubes were heat-treated under different conditions to modify the structure of the TiO_2_ oxide films. In addition, atmospheric pressure plasma was applied to the TiO_2_ nanotubes to change their surface energy and hydrophilicity. This study found that the TiO_2_ nanotubes heat-treated at 400 °C inhibited the adhesion of *S. mutans*, associated with early biofilm formation, and the TiO_2_ nanotubes heat-treated at 600 °C inhibited the adhesion of *P. gingivalis,* which causes peri-implantitis. Applying plasma to TiO_2_ nanotubes heat-treated at 600 °C can also inhibit the adhesion of *S. mutans* and *P. gingivalis*.

## Figures and Tables

**Table 1:** The mean value and standard deviation of TiO_2_ nanotubes.

Group	Diameter (nm)	Thickness (nm)
H0	111.3 ± 2	522.6 ± 4
H400	106.7 ± 1	500.8 ± 4
H600	91.2 ± 1	498.0 ± 7

**Table 2:** The biofilm thickness of *Streptococcus mutans* (*n* = 3).

Group	Biofilm Thickness (μm)		Percentage Thickness Reduction after Plasma Treatment at the Same Heat Treatment (%)
	No Treatment	Plasma Treatment	
H0 ^a,b^	38.1 ± 5	11.1 ± 1 *	70.9
H400 ^c^	22.1 ± 2	17.4 ± 1 *	21.3
H600 ^a,b^	40.9 ± 1	32.9 ± 2 *	19.6

^a, b, c^: Significant at *p* < 0.017 in the column. *: Significant at *p* < 0.05 in the row.

**Table 3:** The biofilm thickness of *Porphyromonas gingivalis* (*n* = 3).

Group	Biofilm Thickness (μm)		Percentage Thickness Reduction after Plasma Treatment at the Same Heat Treatment (%)
	No Treatment	Plasma Treatment	
H0	25.5 ± 1	30.4 ± 4	−19.2
H400	28.4 ± 4	30.9 ± 2	−8.8
H600	26.4 ± 4	24.5 ± 4	7.2

**Table 4:** Parameters of plasma generator.

Parameter	Value
Average working power (W)	300
Voltage (V)	27
Frequency (MHz) *	900
Atmospheric pressure (Torr)	760
Electrode type	Electrodeless
Cooling type	Air cooled
Plasma density	10^15^/cm^3^

*: Variable frequency.

## Data Availability

Not applicable.
